# Effect of Titanium Artifacts on Cholesteatoma in Magnetic Resonance Imaging After Reconstruction of the Middle Ear

**DOI:** 10.3390/jcm14092995

**Published:** 2025-04-26

**Authors:** Christoph J. Pfeiffer, Denis Mihailovic, Hans-Björn Gehl, Lars-Uwe Scholtz, Alexander Kilgue, Conrad Riemann, Dina Voeltz, Ingo Todt

**Affiliations:** 1 Department of Otolaryngology, Head and Neck Surgery, Medical School OWL, Klinikum Bielefeld Mitte, Bielefeld University, Teutoburger Str. 50, 33604 Bielefeld, Germany; 2 Department of Radiology, Medical School OWL, Klinikum Bielefeld Mitte, Bielefeld University, Teutoburger Str. 50, 33604 Bielefeld, Germany; 3 Biostatistics and Medical Biometrics, Medical School OWL, Bielefeld University, Universitätsstr. 25, 33615 Bielefeld, Germany

**Keywords:** cholesteatoma, MRI, non-EPI-DWI sequence, prosthesis

## Abstract

**Background/Objectives**: Surgical removal is the treatment of choice for cholesteatoma control. Depending on the size, the surgery involves partial resection of the ossicular chain and, if necessary, the bony skull base. Titanium foreign materials (prostheses, meshes) can be used to restore sound transmission and to cover larger defects of the skull base. After the operation, recurrence and residual control are necessary. This can be achieved by means of second-look surgery or an MRI examination with a non-EPI DWI sequence. Similarly to other metal implants, artifacts may occur in the image due to the titanium used. In this study, we assessed the magnitude of the MRI hardware differences induced by titanium prostheses and meshes and whether these differences could obscure cholesteatoma detection. **Methods**: 28 MRI examinations (T1-, T2-, non-EPI DWI sequences) in 14 males and 14 females (5.2–92.4 years) after cholesteatoma surgery and single-staged implantation of a PORP, TORP, or titanium mesh were considered. The size of the respective artifacts was measured, and the mean artifact sizes of the respective prosthesis types were compared. A second look surgery was performed in all cases due to the MRI result or clinical findings. Both were also compared. **Results**: Artifacts occurred in all titanium foreign bodies depending on the used MRI sequence (PORP, TORP, Mesh). We found a positive association between the size of the prosthesis and the size of the artifact. All subsequent second-look surgeries confirmed the MRI examinations according to a positive control for the presence of cholesteatoma. The detection rate was 82.1%. All false results were false negatives, and there were no positive results. **Conclusions**: Titanium material-related artifacts might influence the MRI detectability of recurrent cholesteatoma. Small cholesteatoma might be missed by an MRI-based follow-up. This finding supports the reevaluation of single-stage versus staged reconstruction modern approaches.

## 1. Introduction

Cholesteatoma is a non-malignant growing accumulation of squamous epithelium in the middle ear. A cholesteatoma can lead to the destruction of the ossicular chain and the adjacent parts of the temporal bone as well as the skull base [1]. Related destructions and the accompanying infection can cause progressive hearing loss and brain abscesses [2,3].

Surgery is the treatment of choice and can induce further destruction. Surgical main goal is the complete removal of the cholesteatoma [4]. Secondary goals of the operation are to restore sound transmission and the skull base, if affected [5].

In addition to autologous materials such as cartilage, foreign materials such as hydroxyapatite cement, gold, or titanium can likewise be used for reconstruction [6]. Titanium foreign materials (prostheses, meshes) are currently used most frequently [5,7].

Follow-up examinations are needed to detect residual cholesteatoma and recurrences after surgery. This can be performed by means of second-look surgery or a magnetic resonance imagining (MRI) examination with a non-echo planar imaging diffusion weighted (non-EPI DWI) sequence [8,9].

Implants such as ossicular prostheses made of titanium or other metals cause an artifact in MRI [10]. The enlargement of the artifact is dependent on the chosen material, the form, and the size of the implant. Therefore, MRI is a comparatively safe imaging tool for follow-up evaluations, but metal artifacts could lead to its inaccurate prognostics [10,11].

The evaluation of the limitation of cholesteatoma detection by non-EPI DWI sequence-generated MRI artifacts due to titanium foreign material (prosthesis, mesh) is of major relevance, yet it has not been properly addressed.

This study aims to estimate the non-EPI DWI sequence-generated MRI artifact size of titanium foreign material to gain insights into its potential role in cholesteatoma detection.

## 2. Materials and Methods

### 2.1. Patients

In this retrospective study, we screened all patients who underwent a tympanoplasty with resection of a cholesteatoma at our tertiary university hospital between 2020 to 2023.

Inclusion criteria were the implantation of a partial ossicular replacement prosthesis (PORP), a total ossicular replacement prosthesis (TORP), or a titanium mesh during surgery, as well as the performance of an MRI with non-EPI-DWI sequence after implantation. All scans had to be performed on the same machine.

Furthermore, a second operation had to be performed based on the MRI results or clinical findings. The reasons for the second operation were a suspected cholesteatoma on MRI, clinical findings, prosthesis protrusion, or an increasing air-bone gap.

Exclusion criteria were the lack of a follow-up or a second operation and the performance of the MRI on a different system.

A total of 550 cases were screened. Altogether, 27 patients and 28 MRI examinations were included. The mean age of the patients at the time of the MRI was 44.9 years, and the median age was 51.4 years. The sizes of the PORP and TORP varied between patients, and the thickness of the titanium mesh was 0.1 mm.

This study was approved by the local ethics committee (Ärztekammer Westfalen-Lippe, University of Münster Faculty of Medicine, Bielefeld University, Medical Faculty; reference: 2022-314-f-S).

### 2.2. MRI Imaging

In all cases the scans were performed in a 1.5 T MRI system (Achieva, Philips, Best, the Netherlands). The imaging protocol consisted of a T1-, a T2-, an isoDWI-, and a non-EPI DWI sequence.

The parameters for the T1-sequence were time of repetition (TR) 550 ms, time of echo (TE) 10 ms, matrix size (MS) 256 × 256, field-of-view (FOV) 100 mm, slice thickness (ST) 3 mm; for the T2-sequence TR 5058.58 ms, TE 100 ms, MS 512 × 512, FOV 80.21 mm, ST 5 mm; for Non Epi DWI-sequence TR 4134.25 ms, TE 73.67 ms, MS 288 × 288, FOV 104.17 mm, ST 5 mm, B 1000 s/mm^3^; for TR 11,726.62 ms, TE 205.65 ms, MS 128 × 128, FOV 100 mm, ST 3 mm, B 1000 s/mm^3^.

The sizes of the voxels were 0.5 mm in the T1/T2 sequences and 2 mm in the non-EPI DWI sequence.

Before every new surgery, a computer tomography of the temporal bone was performed to clarify the anatomical findings and position of the implanted prosthesis or mesh. The knowledge of the position of the prosthesis or mesh and the anatomical landmarks like the cochlea and the semicircular canals were used to quantify the dimensions of the artifact caused by the implanted prosthesis or meshes in each sequence. The measured artifacts were correlated with the known size of the prosthesis and meshes.

MRI scans were reviewed for evidence of residual or recurrent cholesteatoma. The screening results were compared with the findings from the second surgery performed.

### 2.3. Statistical Analysis

The analysis was performed in R 2024 (RStudio Team, Boston, MA, USA), based on Excel spreadsheets (Microsoft, Redmond, WA, USA). A comparison between the MRI result and the findings of the second surgery was performed. A linear regression analysis was performed to assess the impact of the size and type of implant on the final artifact size. The regression was adjusted to age and sex.

## 3. Results

In this chapter, we describe our results based on the type and size of the prosthesis or mesh as well as the different MRI sequences. We will first present the results and then compare them across the different groups.

During the first operation, a PORP was implanted in 14 cases, a TORP in 11 cases, and a mesh in 3 cases. The sizes of the PORPs and TORPs varied, with 3 and 4 different sizes, respectively (see Table 1). All meshes had a thickness of 0.1 mm.

The time between the operation and the MRI averaged 619 d.

In 17 cases, the MRI showed a cholesteatoma in the non-EPI DWI sequence. In 11 cases, no cholesteatoma was detected.

The second surgery confirmed the results of the MRI in 23 out of 28 cases, giving the positive predictive value of 82.1%. The 5 unconfirmed cases were false positives, where the MRI indicated a cholesteatoma, but surgery showed histopathological verified inflammatory and scar tissue. There were no false negative cases. The sizes of the cholesteatomas found intraoperatively were variable and were not detailed in each of the operative reports. The presence or absence of a cholesteatoma was confirmed by histopathologic examination.

**Figure 1:**
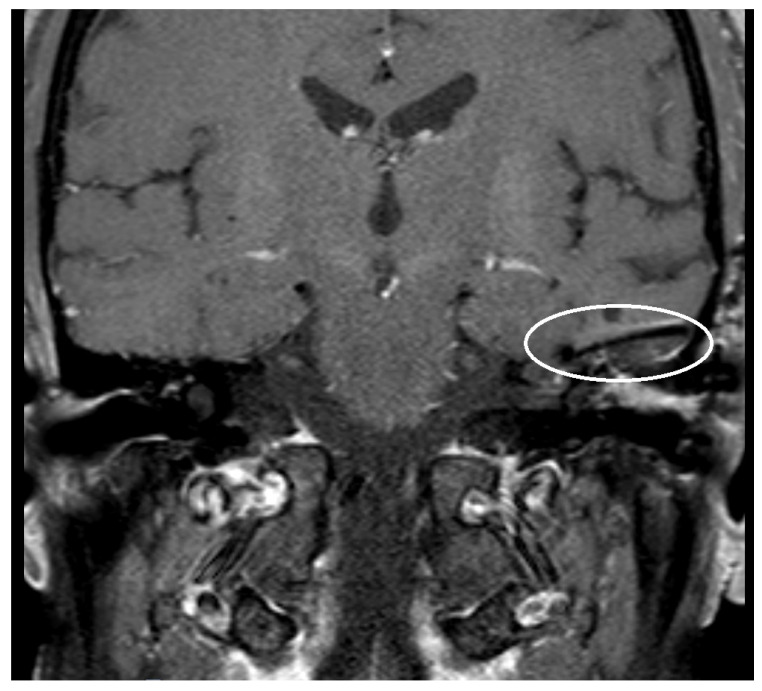
T1 fat-suppressed sequence post-Gadolinium with artifact after implantation of a titanium mesh at the left-sided skull base, as indicated by the circled area.

**Figure 2:**
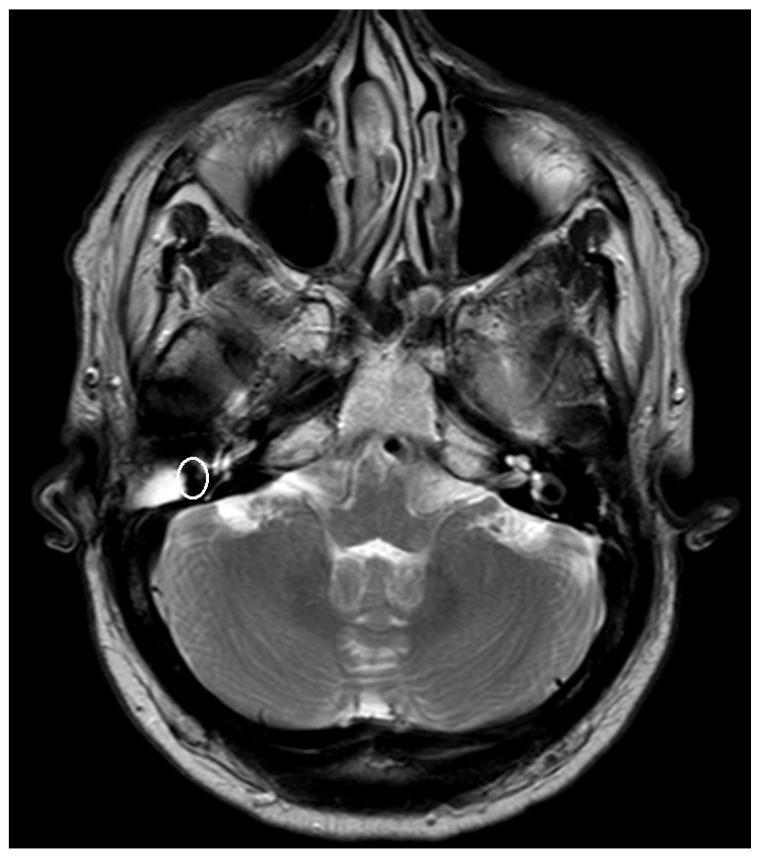
T2 sequence with artifact after the implantation of a PORP in the right-sided middle ear, as indicated by the circled area.

**Figure 3:**
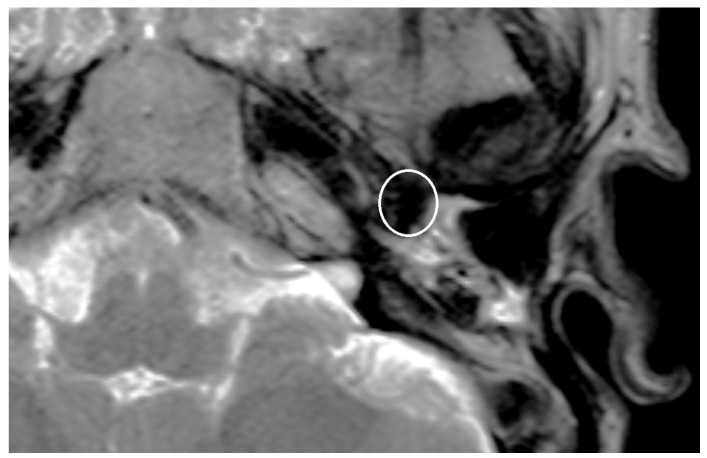
T2 sequence with artifact after the implantation of a TORP at the left-sided middle ear, as indicated by the highlighted region.

An artifact was detected in all MRI examinations (see Figure 1, Figure 2 and Figure 3).

**Figure 4:**
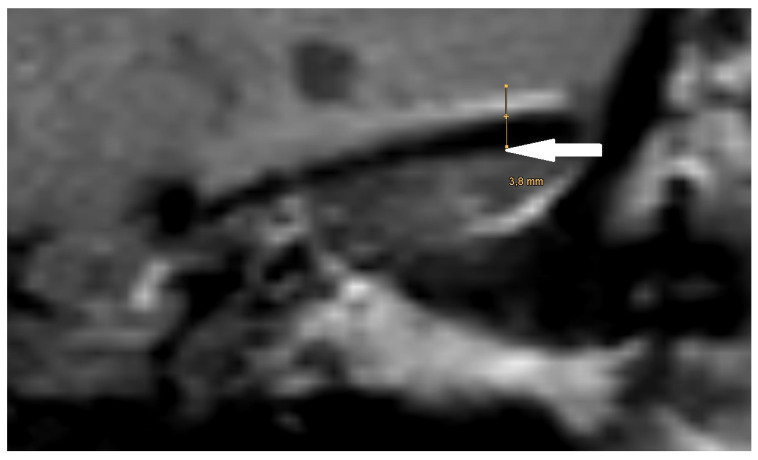
Magnification of the same artifact from Figure 1, with measurement (white arrow).

**Figure 5:**
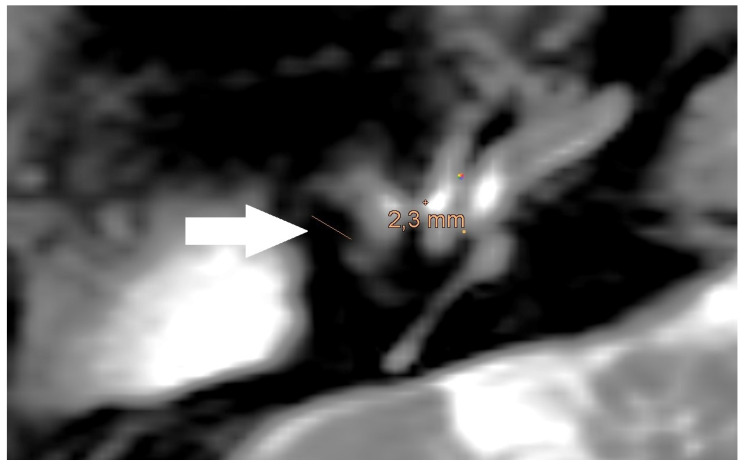
Magnification of the same artifact from Figure 2, with measurement (white arrow).

**Figure 6:**
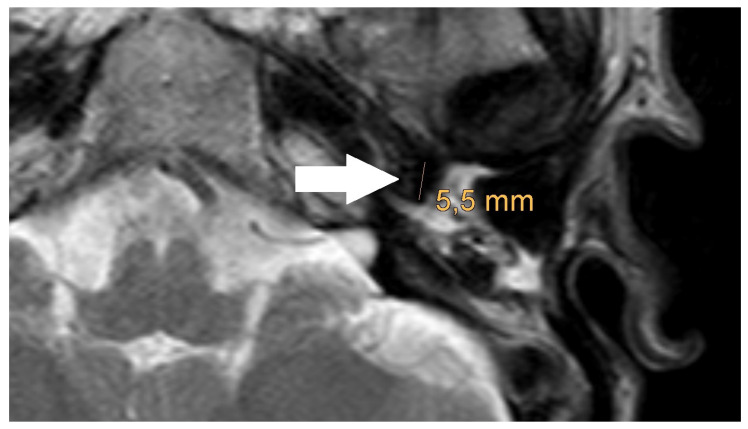
Magnification of the same artifact from Figure 3, with measurement (white arrow).

The artifact measurements, initially performed in the T1- or T2-sequence, showed an average artifact size of 3.1 mm for PORP, 4.7 mm for TORP, and 3.6 mm for meshes (see Table 2 and Figure 4, Figure 5 and Figure 6). The overall average artifact size was 3.8 mm.

The shape of the artifact followed the maximum extension of the respective foreign bodies in ellipsoidal form. For the prostheses, this was the longest distance between the prosthesis plate and the prosthesis base. While the shape of the mesh artifacts followed the maximum extension of the mesh surface and the mesh diameter on the shorter side.

**Figure 7:**
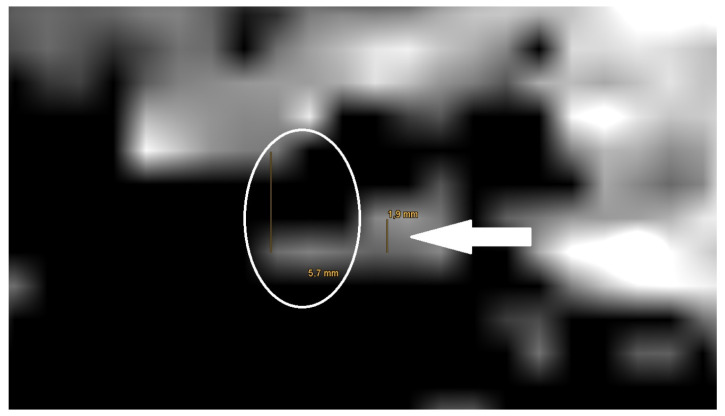
Magnification of the artifact of a mesh on the right side of the lateral skull base in a non-EPI DWI sequence, with measurement (white arrow).

The measurement of the artifact for meshes in the non-EPI DWI sequence showed an average artifact size of 5.8 mm (see Figure 7). Furthermore, the measurements of artifacts for PORP showed an average size 5.4 mm and for TORP 7.2 mm (see Table 2).

The measurement in the non-EPI DWI sequence revealed that the smallest edge length of the voxels in this sequence is 1.9 mm. As a result, the smallest part that can be shown is one voxel (see Figure 7).

**Figure 8:**
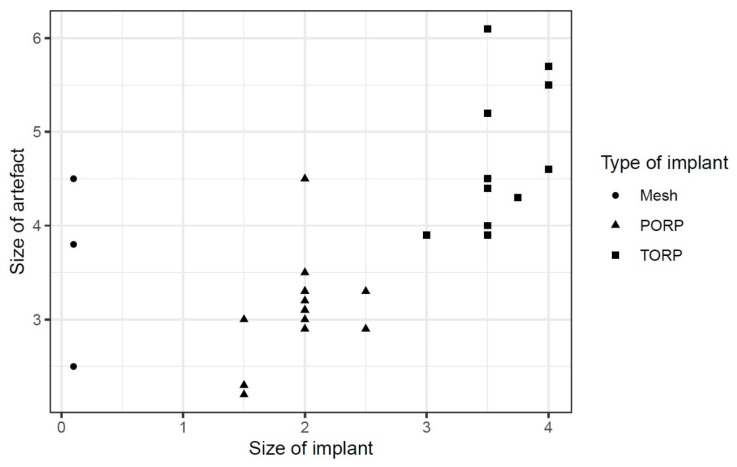
Diagram of relation between size of implant and size of artifact of the different types of implants.

The regression showed that the implant size of PORP and TORP affects the artifact size positively by about 0.876 [95% confidence interval (CI): −0.049; 1.802] (see Figure 8).

Other parameters such as age or gender of the patients showed insignificant influences [age: −0.003, 95% CI: −0.015; 0.009, sex: −0.042, 95% CI: −0.587; 0.503] (see Table 3).

## 4. Discussion

Recurrence control in patients with cholesteatoma is of high importance [12,13,14,15]. There are different concepts of follow-up for cholesteatoma control. While some clinics perform a second-look operation in all cases, other do not see the need for a second-look operation in all cases. A postoperative control with MRI using the non-EPI DWI sequence is a possible method of control without the risk of another anesthesia and surgery [16,17].

### 4.1. Artifact Size and Implant Type

MRI artifacts are an important topic in patients with metal implants. The form and enlargement of the artifact depend on the used metal and the size of the implant [10].

The material used for all protheses and meshes in our study was titanium. Our study shows that a larger size of the used prothesis leads to an increase in the artifact size in MRI. This is consistent with previous studies [11].

The MRI artifact also depends on the magnetic resonance (MR) scanner used for the examination and its magnetic field strength [11]. In all examinations in this study, the same MR scanner was used.

Another factor influencing the artifact is the MRI sequence. The local resolution varies between different MRI sequences. A non-EPI DWI sequence has a higher detection rate for cholesteatoma than other MRI sequences [15]. However, T1 and T2 sequences have a higher resolution than the non-EPI DWI sequence. The resolution of different MRI sequences has an impact on cholesteatoma detection [18].

In our study, the detection rate in the non-EPI DWI sequence (82.1%) was lower than in other studies. In these studies, a reconstruction with prostheses was not mentioned or ruled out [16,17,19]. The false positive results were associated with scarring and inflammation. The presence of an Otitis media with effusion (OME) can have an impact on the recognition of a false positive signal in the non-EPI DWI sequence. Additionally, the lower rates of OME during the COVID-19 pandemic could have influenced the false positive rates in the non-EPI DWI sequence [20].

### 4.2. Consequences of MRI-Based Surveillance

The detection rate of a cholesteatoma also depends on the presence of a prosthesis and the proximity of the cholesteatoma to the prosthesis. In our study, the prostheses caused artifacts of at least 2.2 mm in the T1/T2 sequences. In the non-EPI DWI sequence, the voxel size is 1.9 mm, which is larger than in T1/T2 sequences with 0.5 mm. Therefore, to detect a cholesteatoma, it must be larger than 1.9 mm in the absence of a prosthesis and larger than the artifact of the prosthesis in the presence of a prosthesis. A smaller cholesteatoma would be covered by the artifact of the prosthesis. Our findings are consistent with previous studies [18].

There were false positive examination results in the MRI in our study but no false negatives. Other studies show similar results. Thus, false positive results can occur due to inflammation in the middle ear or cartilage grafts [18,21].

In previous studies, especially small cholesteatomas were found in second-look operations, even when the MRI showed no signs of a cholesteatoma [14,22]. This is similar to our assumptions for the non-EPI DWI sequence because of the limitations of the voxel size, which is also consistent with former studies [18].

Our study’s limitations include the small number of patients and the lack of artifact predictability. The study used a retrospective, single-center design. The characterization of the artifacts was performed on a single MRI system. Other MRI systems may show different results.

A follow-up with MRI examinations should be considered to detect a growing cholesteatoma [14,22].

### 4.3. Clinical Decision-Making in Ossicular Reconstruction

The thought of small cholesteatomas should be considered in the assessment of ossicle reconstruction in a single stage or later operation. The decision to perform a single-stage ossicle reconstruction with cholesteatoma excision affects the detection of recurrent cholesteatomas and may be a reason for a second-look operation. The use of new high-resolution imaging techniques can also lead to lower rates of residual or recurrent cholesteatoma [23].

All in all, the detection of a cholesteatoma depends on several factors. Foreign material, especially metal used for reconstruction, affects the size of the artifact in the MRI. The chosen MRI sequence also influences the artifact. The MRI sequence determines the size of the voxels in the image, with the presumptive border of detection being around 2 mm.

Under these circumstances, the size of the cholesteatoma influences its detectability. Larger lesions are easily detected with MRI, but small lesions near implants may be overlooked. These considerations should be taken into account when deciding on immediate or delayed hearing reconstruction during ear surgery for cholesteatoma removal.

## 5. Conclusions

Artifacts associated with titanium implants could affect the detectability of recurrent cholesteatoma on MRI. Titanium-related MRI artifacts may conceal cholesteatomas smaller than 2 mm in size. Therefore, the decision between single or two-stage reconstruction approaches after cholesteatoma surgery needs careful consideration. A prospective multicenter study is warranted to further explore these findings.

## Figures and Tables

**Table 1:** Descriptive statistics.

Variable	Mesh	PORP	TORP
**Age**	38.77 (sd = 20.60, min = 26.64, max = 62.56)	47.62 (sd = 23.22, min = 5.20, max = 78.51)	43.03 (sd = 25.83, min = 12.53, max = 92.45)
**Sex**			
m	1 (33%)	7 (50%)	6 (55%)
w	2 (67%)	7 (50%)	5 (45%)
**Size of artifact**	3.60 (sd = 1.01, min = 2.50, max = 4.50)	3.11 (sd = 0.54, min = 2.20, max = 4.50)	4.74 (sd = 0.77, min = 3.90, max = 6.10)
**Size of implant**			
0.1	3 (100%)	0 (0%)	0 (0%)
1.5	0 (0%)	3 (21%)	0 (0%)
2	0 (0%)	9 (64%)	0 (0%)
2.5	0 (0%)	2 (14%)	0 (0%)
3	0 (0%)	0 (0%)	1 (9.1%)
3.5	0 (0%)	0 (0%)	6 (55%)
3.75	0 (0%)	0 (0%)	1 (9.1%)
4	0 (0%)	0 (0%)	3 (27%)

**Table 2:** Average artifact size by sequence and implant type.

	Prosthesis Size	Artifact Size	
		T1/T2	Non-EPI DWI Sequence
PORP	2.0 mm	3.1 mm	5.4 mm
TORP	3.6 mm	4.7 mm	7.2 mm
Mesh	0.1 mm	3.6 mm	5.8 mm

**Table 3:** Regression analysis.

Regression Analysis				
	Estimate Std.	Error	t Value	Pr (>|t|)
Intercept	1.547	1.031	1.501	0.149
Implant_size	0.876	0.444	1.975	0.062
implantTORP	0.162	0.765	0.212	0.834
age	−0.003	0.006	−0.494	0.627
sexw	−0.042	0.261	−0.162	0.873

## Data Availability

The original data are available from the corresponding author upon request.

## Abbreviations

The following abbreviations are used in this manuscript:

MRI	magnetic resonance imagining
non-EPI DWI	non-echo planar imaging diffusion weighted
PORP	partial ossicular replacement prosthesis
TORP	total ossicular replacement prosthesis
CI	confidence interval
MR	magnetic resonance
OME	Otitis media with effusion
